# Psychosocial interventions for healthcare workers during the COVID-19 pandemic: rapid review and meta-analysis

**DOI:** 10.1007/s10354-023-01013-9

**Published:** 2023-05-16

**Authors:** Manuela Hoedl, Selvedina Osmancevic, Nina Thonhofer, Lea Reiter, Daniela Schoberer

**Affiliations:** Institute of Nursing Science, Medical University of Graz, Universitaetsplatz 4/3, 8010 Graz, Austria

**Keywords:** Mental health, Occupational health, Pandemic, SARS-Cov-2, Stress, Psychische Gesundheit, Gesundheit am Arbeitsplatz, Pandemie, SARS-CoV‑2, Stress

## Abstract

This rapid review was conducted by following a predefined protocol developed by the Cochrane Rapid Reviews Methods Group. A total of 172 potential reviews and 167 primary studies of interest were found. AMSTAR II was used to assess the quality of the included reviews and the JBI Checklist for Randomized Controlled Trials for primary studies. Overall, four studies were included in this review. The study quality ranged from 5 to 12 out of 13 possible stars. No robust evidence was found that psychosocial interventions can reduce psychological distress. No significant effect could be found regarding post-traumatic stress. Two studies on anxiety were identified, one which showed an effect and another which did not. The psychosocial intervention had no beneficial effect on burnout and depression, whereas providing a mindfulness- or relaxation-based intervention resulted in a significant improvement in sleep quality. By considering the secondary results and results of previous reviews, a combination of training and mindfulness seems to be beneficial for decreasing anxiety and stress in home care workers. In summary, the evidence-based recommendations are still limited and, therefore, more evidence is needed to make a general statement of high certainty about the effects.

**Supplementary Information:**

The online version of this article (10.1007/s10354-023-01013-9) contains supplementary material, which is available to authorized users.

## Abstract (de)

Dieses Rapid Review wurde nach einem vordefinierten Protokoll durchgeführt, das von der Cochrane Rapid Reviews Methods Group entwickelt wurde. Insgesamt wurden 172 potenzielle Reviews und 167 Primärstudien von Interesse erfasst. Das Instrument AMSTAR II wurde zur Bewertung der Qualität der in die Auswertung eingeschlossenen Reviews und die JBI-Checkliste (Joanna Briggs Institute, Aidelaide, Australien) für randomisierte kontrollierte Studien zur Beurteilung der Primärstudien verwendet. Insgesamt wurden 4 Studien in die Analyse einbezogen. Die Qualität der Studien reichte von 5 bis 12 von 13 möglichen Sternen. Es konnten keine stichhaltigen Beweise gefunden werden, dass psychosoziale Interventionen die psychische Belastung verringern. In Bezug auf posttraumatischen Stress konnte keine signifikante Wirkung festgestellt werden. Es wurden 2 Studien zum Thema Angst ermittelt, in einer davon wurde eine Wirkung nachgewiesen, in der anderen jedoch nicht. Die psychosoziale Intervention hatte keinen positiven Effekt auf Burnout und Depression, während eine achtsamkeits- oder entspannungsbasierte Intervention zu einer signifikanten Verbesserung der Schlafqualität führte. Betrachtet man die sekundären Ergebnisse und die Ergebnisse früherer Untersuchungen, so scheint die Kombination aus Training und Achtsamkeit für die Verringerung von Angst und Stress bei Pflegekräften in der ambulanten Pflege von Vorteil zu sein. Zusammenfassend lässt sich sagen, dass die evidenzbasierten Empfehlungen immer noch begrenzt sind und daher mehr Studien benötigt werden, um mit hoher Sicherheit eine allgemeine Aussage über die Auswirkungen zu treffen.

## Introduction

All over the world, healthcare systems are dealing with the physical and psychological consequences of the pandemic [1]. In all societies, psychological consequences of the restrictive interventions applied to prevent spread of the virus or consequences of becoming infected with SARS-CoV‑2 commonly occur.

Up to 25% of the general population reported experiencing clinically relevant anxiety symptoms [2, 3]. These studies also indicated that being female is significantly associated with a higher level of fear and anxiety about COVID-19 than being male [2, 3].

In addition to the psychological consequences of the pandemic, another recent article even referred to the COVID-19 pandemic as a traumatic stressor [4]. The authors concluded that COVID-19 is a traumatic stressor event that can consequently lead to serious mental health problems, such as post-traumatic stress disorder (PTSD) [4]. Post-traumatic stress disorder is a common psychopathological consequence of exposure to traumatic events, including symptoms such as anxiety, sleep difficulties, and irritability [5].

In their systematic review, Zhang et al. reported observing a 15% prevalence of PTSD in the general population during the COVID-19 pandemic [6]. PTSD not only concerns the general population. Members of the healthcare workforce (HCW) and especially those who worked on COVID-19 wards experienced exceptionally stressful situations on a daily basis, which may lead to even higher rates of PTSD.

A recent systematic review and meta-analysis concluded that 26% of HCWs had experienced PTSD as a result of the SARS, H1N1, Ebola, and Zika pandemics [7]. A systematic review was performed by d’Ettorre et al. (2021) on topics and interventions related to post-traumatic stress symptoms in HCWs during the current pandemic. They reported that 2.1–73.4% of HCWs reported experiencing post-traumatic stress symptoms [8]. In addition, a review of the prevalence of trauma- and stressor-related symptoms experienced by HCWs during the COVID-19 pandemic found a trauma-related stress prevalence of up to 35% [9]. These authors highlighted in their findings the fact that women, nurses, and frontline workers were most strongly affected by trauma-related stress [9].

Within the Austrian healthcare system, 41% of hospital nurses felt a high to very high level of psychological distress as a result of the COVID-19 pandemic, including symptoms such as anxiety, worries, or insomnia. More than two thirds of Austrian nursing staff, also including nursing aids as well as students, experienced a moderate to high level of stress [10]. In another study, nurse managers from Austria referred to a loss of image, a reduction in job satisfaction, and mental stress [11].

On the other hand, the healthcare and especially nursing staff shortage presents a challenge worldwide. As an example, the International Council of Nurses recently conducted a survey and reported that 20% of the respondents indicated that an increasing number of nurses are leaving the profession as a result of the pandemic [12]. The authors also estimated that a shortage of nearly 13 million nurses worldwide would occur in the future [12]. For the Austrian situation, a representative survey revealed that 86% of nurses stated that their work situation in the hospital had worsened to strongly worsened due to the pandemic [13]. In addition, 45% of nurses thought about leaving their profession and, moreover, 5% were planning and implementing their career change at the time of the survey [13]. This is underlined by the report on the state of the world’s nursing from the WHO, which urgently recommended conducting investigations on education, jobs, and leadership internationally [14].

Overall, the literature indicates that the COVID-19 pandemic was and is a major traumatic stressor, leading to an increase in the work burdens placed on HCWs and nursing staff, specifically due to the current and future nursing staff shortage. Therefore, interventions have to be set to increase job retention and facilitate recruitment, such as financial investments or psychosocial interventions to reduce or minimize the psychological impact of pandemics on the healthcare workforce. A systematic review revealed that only cross-sectional studies have been carried out on strategies to manage post-traumatic stress symptoms in HCWs during the COVID-19 pandemic [8]. These authors identified occupational training and the improvement of social support at work as primary strategies used to manage post-traumatic stress symptoms in HCWs during the pandemic. However, the effects of applying these strategies could not be measured with these study designs. Although evidence is accumulating for the impact of the COVID-19 pandemic on HCWs, studies on the effect of psychosocial interventions among HCWs are lacking.

Interventions commonly used to increase mental health in HCWs might also not work because the pandemic itself is a traumatic stressor event.

As an added complication, we currently have no evidence for the effects of psychosocial interventions in HCWs that are restricted to the COVID-19 pandemic. Managers are struggling to answer the question of how best to support HCWs in pandemics in order to reduce their stress and stress-related symptoms. For these reasons, this study was carried out to investigate the effectiveness of psychosocial interventions on the ward or at the institutional level among HCWs during the COVID-19 pandemic.

## Patients, materials, and methods

This rapid review was conducted by following a predefined protocol, which was peer reviewed and reflected upon by the authors of this paper. In a first step, two authors developed a research question and defined the intervention as well the outcomes, which were reviewed by the other authors. In a second step, specific inclusion and exclusion criteria with regard to the design, methodology, population, setting, interventions, and outcomes were set by two authors, which were again critically appraised by the other authors.

We defined psychosocial interventions as “interventions that have their primary mode of action through psychological or social processes. Such interventions include, for instance, direct therapeutic work, health education and social support” [15]. We used this definition, because the COVID-19 pandemic is considered to be a major traumatic stressor. In addition, De Silva et al. (2009) focused on traumatic physical injuries that led to mental health problems, such as depression, anxiety, or PTSD; these problems are also experienced by HCW affected by the COVID-19 pandemic [15].

### Search strategy

The search strategy was applied in two steps. The first search step was carried out to identify systematic reviews from which the data could be extracted. The second search step was conducted to identify primary studies which had been published since the search for systematic reviews was carried out.

The databases CINAHL (Cumulative Index to Nursing and Allied Health Literature), PubMed, and CDSR (Cochrane Database of Systematic Reviews) were searched to identify systematic reviews. The search for primary studies was conducted in the CINAHL, PubMed, and CENTRAL databases. Both search strategies involved the use of specific keywords and their respective Medical Subject Headings (MeSH)/Subject Headings. Even though we did not specifically search for grey literature, we used CENTRAL database, which included clinical study registers. If we found a suitable clinical study register (e.g., a protocol), we looked to see if there was already a preprint.

No language restrictions were applied (see Supplementary Information Tables 1 and 2). Systematic reviews needed to have been published after 1 January 2020, which was denoted as the beginning of the COVID-19 pandemic. Any additional primary studies that were found had to have been published after 24 June 2021. Screening of titles, abstracts, and full-text papers was conducted separately by two independent researchers, and disagreements were resolved through discussion.

The inclusion criteria for this study were: a) population—HCWs who provided direct patient care; b) intervention—psychosocial interventions were performed within the work setting; c) outcomes—stress, depression, anxiety, sleep disorder, sleep disturbance, insomnia, burnout, distress, anxiety, psychological distress; and d) setting—hospitals and long-term care institutions.

Systematic reviews of epidemiological studies, syntheses that lacked a systematic approach, and reviews of national guidelines and qualitative studies were excluded. Studies that focused on outpatient and home care were also excluded. Reviews in which HCWs such as nursing managers did not perform direct care were excluded unless the data were presented separately (see Supplementary Information Table 3). We also excluded studies where interventions had been adopted by an isolated individual or were not offered on a ward or an institutional basis.

### Data extraction and quality assessment

One researcher extracted the following data from the reviews: author; review characteristics such as intervention, population, endpoints, and setting of interest; and information about the search strategy and primary studies of interest. The following data were extracted from the primary studies: author; study design; population; setting; type and details of intervention; and outcome measures including the used instruments. The quality of the primary studies was also extracted from the reviews. A second researcher checked the data extraction for comprehensiveness.

We used AMSTAR II to assess the quality of the included reviews (see Supplementary Information Table 4). The primary studies which were identified in the second search step were critically appraised using the JBI Checklist for Randomized Controlled Trials [16] (see Supplementary Information Table 4). Both assessments were performed independently by two researchers. Any disagreements were resolved through discussion. We decided to exclude the questions on the meta-analysis in AMSTAR II, because we were interested in detecting relevant primary studies.

### Data synthesis

Study results were grouped according to the stress and stress-related outcomes, and primary results were presented as mean differences and *p*-values. If at least two studies examined similar interventions and outcomes, the results were synthesized [17]. The random effects model was used as no fixed effect can be assumed due to expected diverse interventions, which influences the study effect estimates [18]. Heterogeneity was assessed using the I^2^ statistic. We calculated the pooled standardized mean difference with 95% confidence intervals (CI) by applying the inverse variance method. Meta-analyses were performed with RevMan 5.4.1 (The Cochrane Collaboration, London, UK) [19].

## Results

**Fig. 1:**
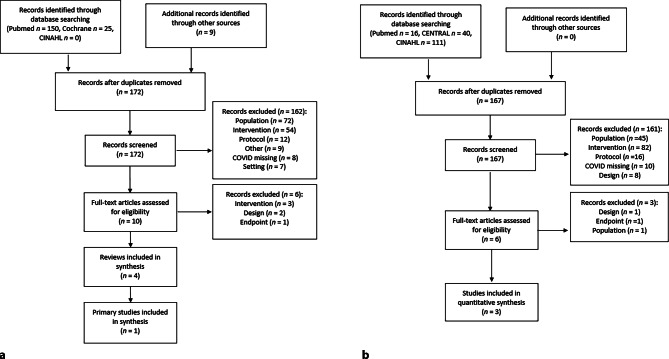
Search strategy for systematic reviews (**a**) and primary studies (**b**)

We initially identified 172 potential reviews of interest (Fig. 1). After performing title and abstract screening, we narrowed this pool down to 10 relevant references and subsequently screened the full-text papers.

Two primary studies were then found which met our inclusion criteria [20, 21], which were cited in one review [22]. One of these primary studies had to be excluded [20], because the study results were not traceable and the authors failed to respond to repeated attempts to contact them.

We identified 167 potential primary studies of interest. Six of these were subsequently deemed eligible for full-text screening, which resulted in the inclusion of three further studies [23–25]. Overall, we included four studies in this review, one of which resulted from the search for reviews and the remaining three of which were found during the search for primary studies.

### Study characteristics and quality assessment

Supplementary Information Table 5 shows the quality of the included studies. All of these studies were carried out as randomized controlled trials [23–25], with the exception of the study by Zhou et al. (2020) which used a before–after design [21]. The study participants were all HCWs, most of whom worked in a hospital (Supplementary Information Table 6). The sample sizes reported in these studies varied from 41 participants [24] to 482 participants [23], with 749 participants included in total.

The primary outcomes measured in these studies were psychological distress/PTSD, anxiety, depression, burnout, and insomnia/sleep. In total, seven different surveys and scales were applied to measure the outcomes (Supplementary Information Table 6). Anxiety was measured with the DASS-21 subscale for anxiety (depression, anxiety, stress scale) [23] and the self-rating anxiety scale (SAS) [21]. Depression was measured using the DASS-21 subscale for depression [23] and the self-rating depression scale (SDS) [21]. Post-traumatic stress and psychological distress were measured in one study using the DASS-21 stress subscale as well as the Davidson Trauma Scale (DTS) [23].

Fiol-DeRoque et al. (2021) also measured burnout using the Maslach Burnout Inventory-Human Services Survey (MBI-HSS) [23]. Insomnia/sleep were the most frequent outcomes, which were measured in three studies [23–25]. These outcomes were surveyed using the Pittsburgh Sleep Quality Index (PSQI) [24, 25] or the Insomnia Severity Index (ISI) [23].

The ratings obtained from the application of the JBI Checklist for Randomized Controlled Trials ranged from 5/13 [24] to 12/13 [23] (see Supplementary Information Table 5).

### Performed psychosocial interventions in the COVID-19 pandemic

Three studies included aspects of mindfulness in their interventions [21, 23, 24].

Fiol-DeRoque et al. (2021) explored the effectiveness of using a psychoeducational app called PsyCovidApp (Instituto de Investigación Sanitaria Islas Baleares, Palma, Spain), which was created by mental health experts [23]. The app content was based on aspects of cognitive-behavioral therapy and mindfulness.

The intervention group had access to the app for 14 days, during which they received daily notifications or were asked to answer short questionnaires that had been designed to encourage improvements in lifestyle, stress, social support, or emotional skills.

Nourian et al. (2021) studied the effectiveness of mindfulness-based stress reduction (MBSR). For this intervention, the intervention group received audio and video files with instructions for performing meditation and yoga exercises. The group also received texts on the benefits of mindfulness, and audio and video files with mind exercises guided by professionals. The group was asked to perform the MBSR program for at least 1 hour a day, 6 days a week, over a period of 7 weeks.

Another study that included mindfulness was carried out by Zhou et al. (2020). In this study, nurses were provided with on-site, online, and drill training as well as psychological counselling. The online training consisted of videos, graphics, and textual information about hygiene measures, ways to prevent infection, and ways to diagnose and take care of COVID-19 patients. As psychological support, participants received counselling and instruction on mindfulness decompression therapy from psychologists to encourage mindfulness.

The remaining intervention, which was performed in the study of Thimmapuram et al. (2021), was a heart-based meditation [25]. The participants in this study received audio files with guided meditations that encouraged them to focus on their heart. The meditations had to be performed in the mornings and evenings, lasting approximately 6 min each time. Depending on the time of day, the meditation was designed to encourage relaxation or to improve the sense of self. Participants took part in the program for 4 weeks.

### Effects of the interventions

Stress and stress-related outcomes could be categorized into psychological distress, anxiety, burnout, depression, and insomnia/sleep quality (Supplementary Information Table 7).

In Fiol-DeRoque et al. (2021), the DASS-21 overall score was reported, including depression, anxiety, and stress [23]. This score did not change significantly when the PsyCovidApp was used (MD: −0.04; CI: −0.11–0.04; *p* = 0.15).

#### Psychological distress/PTSD

Fiol-DeRoque et al. (2021) was the only study to explore the effect of an intervention on psychological stress and PTSD [23]. The DASS-21 subscale for stress was used and the results indicate that use of the PsyCovidApp could be correlated with a marginally non-significant reduction in the stress score (MD: 0.06; CI: −0.14–0.01; *p* = 0.05). No significant effect could be found regarding the impact of the PsyCovidApp on PTSD measured with the DTS (MD: 0.00; CI: −0.06–0.07; *p* = 0.47).

#### Anxiety

Two studies investigated the outcome anxiety [21, 23]. Fiol-DeRoque et al. (2021) applied the anxiety subscale from the DASS-21 and showed that the use of the app resulted in a non-significant reduction of 0.04 points (MD: −0.04; CI: −0.12–−0.04; *p* = 0.17). Zhou et al. (2020) used the SAS and showed that the skills training and psychological support significantly decreased the participants’ anxiety (*p* = 0.019) [21].

#### Burnout

Burnout was only measured by examining the effect of the use of the PsyCovidApp [23]. To measure burnout, the MBI-HSS was applied. This tool consists of subscales of emotional exhaustion, personal accomplishment, and depersonalization. The intervention showed no effect on any of the three (*p* = 0.39, *p* = 0.12, *p* = 0.36).

#### Depression

The outcome depression was measured in the studies by Fiol-DeRoque et al. (2021) and Zhou et al. (2020). Fiol-DeRoque et al. (2021) showed that the mean score for the DASS-21 depression subscale did not change due to the intervention (MD: 0.00; CI: −0.07–0.08; *p* = 0.47) [23]. Neither study found any significant effect of the tested interventions on depression as measured with the DASS-21 depression subscale (*p* = 0.47) and the SDS (*p* = 0.306) [21].

#### Sleep

**Fig. 2:**
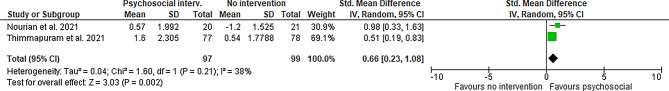
Psychosocial intervention versus no intervention—outcome: improvement in sleep quality as measured by the Pittsburgh Sleep Quality Index

Three studies examined the effects of psychosocial interventions on sleep [23–25]. Two of these used the same endpoint (sleep quality) and instrument (PSQI) [24, 25], which was why a meta-analysis was performed with these studies. The pooled effect (Fig. 2) revealed a significant improvement in sleep quality by implementing the psychosocial interventions, with low levels of heterogeneity (SMD = 0.66; 95%CI = 0.23–1.08; *p* = 0.002; I^2^ = 38%). Fiol-DeRoque et al. (2021) examined the effect of the PsyCovidApp on insomnia with the tool ISI. Both groups experienced a reduction in insomnia during the study period; however, the size of the reduction did not differ significantly between the intervention and the control group (*p* = 0.38)[23].

### Secondary outcomes

The level of acceptance of an intervention can be indicated by the dropout and lost-to-follow-up rates. Only the studies on the heart-based meditation and the PsyCovidApp stated the number of participants who were lost to follow-up [23, 25]. Both studies had higher dropout rates in the intervention group than in the control group. Regarding usability, it is important to note that the PsyCovidApp had a high score when measuring the usability of the intervention (87.21/100; SD: 12.65). In addition, over 90% of the intervention group asked to have access to the app again [23].

In the study by Zhou et al. (2020), participants were asked to rate how useful they considered each type of on-site and online training. The combination of online and on-site training was considered to be useful for all types of training; however, this result was only significant for drill (*p* = 0.002) and theoretical training (*p* = 0.042).

Furthermore, when analyzing the effect of the online training forms (text, graphics, video), online texts (*p* = 0.042) and videos (*p* = 0.040) provided in the course of operational training proved to be significantly effective. Further information can be found in Supplementary Information Table 7.

## Discussion

Based on the number of studies included in this review, we can conclude that the knowledge about the effectiveness of psychosocial interventions for HCWs during COVID-19 is still limited.

We found no robust evidence that psychosocial interventions can reduce psychological distress or PTSD. However, a recent systematic review on generally dealing with infectious diseases found that training and education on infectious diseases can be beneficial, not only for dealing with but also for preventing psychological distress [26]. Similar results were found in a systematic review from 2019, which measured the effect of mindfulness-based interventions on psychological distress in nurses. Six out of the nine included studies showed a decrease in the nurses’ stress levels [27].

The authors of a before–after study reported observing a significant reduction in the perceived level of stress in 52 nurses (*p* = 0.01) as a result of a 4-hour mindfulness workshop [28]. However, we identified only one study that described a very specific intervention: the PsyCovidApp [23].

The review by Ghawadra et al. (2019) also reported a decrease in depression and anxiety as a result of providing mindfulness-based interventions [27]. These findings are underlined by the results of the included study by Zhou et al. (2020), which showed that providing nurses with training and psychological support is an effective intervention to significantly decrease their anxiety [21]. Although this study was appraised as a good-quality study, it was not a controlled study, and many other aspects (e.g., reduced uncertainty due to the persistence of the pandemic) may have influenced the study results.

Our review results indicate that significant improvements can occur in sleep quality when a mindfulness- or rather relaxation-based intervention is provided. Nourian et al. (2021) presented data on sleep quality for each component of the PSQI [24], demonstrating a significant improvement in subjective sleep quality and sleep latency. Thimmapuram et al. (2021) presented only the overall PSQI score [25]. It would have been interesting to see the results for each component of the PSQI in order to gain a better understanding of the intervention and to see which components of sleep show the greatest influence. It is important to note that the pooled results on sleep quality are based on data from only two low-quality studies and, therefore, these should not be overinterpreted.

Even though three of our studies addressed interventions which included aspects of mindfulness, the interventions still differed from one another. In addition, the measured outcomes were not the same or, if they were the same, different instruments and surveys were used for the measurement. This consideration makes it difficult to analyze and compare the effects of these mindfulness-based interventions. We also have to mention here that even though our topic was psychosocial interventions, specific databases for psychological science such as PsycINFO were not searched.

Certain components of our study outcomes show that mindfulness interventions can be significantly effective, and the published literature indicate that these types of interventions may be beneficial for HCW. In addition to mindfulness programs, several other psychological interventions have already been generally recognized by previous studies as ways of preventing and dealing with the psychological outcomes of outbreaks such as COVID-19 [26, 29, 30]. However, interventions that are considered as psychological interventions must be clearly defined to execute targeted actions to protect HCW from further negative psychological outcomes.

## Conclusion

This study explored the effects of various psychosocial interventions on HCWs. Certain components of the measured outcomes showed significant improvement after a psychosocial intervention was used. However, more evidence is needed to make a general statement of high certainty about the effects of these psychosocial interventions, as these varied in execution and measured outcomes. Therefore, psychosocial interventions within the context of our research question must considered separately until further studies are published on this topic. The secondary results and the results of previous reviews indicate that the combination of training and mindfulness may be beneficial to decrease anxiety and stress in HCWs.

### Supplementary Information

Supplementary Information Tables 1 to 7
Table 1. Search terms used for literature search
Table 2. Search strings for the databases (exemplary for systematic reviews)
Table 3. Excluded systematic reviews
Table 4. AMSTAR quality assessment of the included systematic reviews
Table 5. JBI quality assessment of included primary studies
Table 6. Characteristics of the included studies
Table 7. Results of the included studies according to stress-related outcomes

## References

[1] Evans RA (2021). Physical, cognitive, and mental health impacts of COVID-19 after hospitalisation (PHOSP-COVID): a UK multicentre, prospective cohort study. Lancet Respir Med.

[2] Fitzpatrick KM, Harris C, Drawve G (2020). Fear of COVID-19 and the mental health consequences in America. Psychol Trauma.

[3] Moghanibashi-Mansourieh A (2020). Assessing the anxiety level of Iranian general population during COVID-19 outbreak. Asian J. Psychiatry.

[4] Bridgland VME (2021). Why the COVID-19 pandemic is a traumatic stressor. PLoS ONE.

[5] Shalev A, Liberzon I, Marmar C (2017). Post-traumatic stress disorder. N Engl J Med.

[6] Zhang L (2021). The prevalence of post-traumatic stress disorder in the general population during the COVID-19 pandemic: a systematic review and single-arm meta-analysis. Psychiatry Investig.

[7] Yuan K (2021). Prevalence of posttraumatic stress disorder after infectious disease pandemics in the twenty-first century, including COVID-19: a meta-analysis and systematic review. Mol Psychiatry.

[8] d’Ettorre G (2021). Post-traumatic stress symptoms in healthcare workers dealing with the COVID-19 pandemic: a systematic review. Int J Environ Res Public Health.

[9] Benfante A (2020). Traumatic stress in healthcare workers during COVID-19 pandemic: a review of the immediate impact. Front Psychol.

[10] Hoedl M, Bauer S, Eglseer D (2021). Influence of nursing staff working hours on stress levels during the COVID-19 pandemic: a cross-sectional online survey. HeilberufeScience.

[11] Schoberer D (2023). Occupational relationships and working duties of nursing management staff during the COVID-19 pandemic: a qualitative analysis of survey responses. J Adv Nurs.

[12] International Council of Nurses. The global nursing shortage and nurse retention. 2021. https://www.icn.ch/sites/default/files/inline-files/ICN%20Policy%20Brief_Nurse%20Shortage%20and%20Retention_0.pdf. Accessed 11 May 2023.

[13] Gferer A, Gferer N. Gesundheits- & Krankenpfleger*innen während der COVID-19 Pandemie in Österreich: Arbeitssituation und Gedanken an einen Ausstieg aus dem PFlegeberuf. 2021. https://www.oegkv.at/fileadmin/user_upload/Aktuell/2021/OEGKV-Homepage_Gferer___Gferer_GuK-C19-Studie_08.06.21.pdf. Accessed 11 May 2023. 10.1007/s00735-021-1378-6 PMC8445791 34548761

[14] WHO (2021). State of the world’s nursing report.

[15] De Silva M (2009). Psychosocial interventions for the prevention of disability following traumatic physical injury. Cochrane Database Syst Rev.

[16] Tufanaru C, Aromataris E, Munn Z (2020). Chapter 3: systematic reviews of effectiveness. JBI manual for evidence synthesis.

[17] Deeks J, Higgins J, Thomas J, Chandler J, Cumpston M (2017). Chapter 9: Analysing data and undertaking meta-analyses. Cochrane Handbook for Systematic Reviews of Interventions version 5.2.0.

[18] Schwarzer G, Carpenter JR, Rücker G (2015). Meta-analysis with R.

[19] The Cochrane Collaboration (2021). Review Manager Web (RevMan Web).

[20] Cai Z (2020). Nurses endured high risks of psychological problems under the epidemic of COVID-19 in a longitudinal study in Wuhan China. J Psychiatr Res.

[21] Zhou M (2020). Research on the individualized short-term training model of nurses in emergency isolation wards during the outbreak of COVID-19. Nurs Open.

[22] Zaçe D (2021). Interventions to address mental health issues in healthcare workers during infectious disease outbreaks: a systematic review. J Psychiatr Res.

[23] Fiol-DeRoque M (2021). A mobile phone-based intervention to reduce mental health problems in health care workers during the COVID-19 pandemic (PsyCovidapp): randomized controlled trial. JMIR Mhealth Uhealth.

[24] Nourian M (2021). The impact of an online mindfulness-based stress reduction program on sleep quality of nurses working in COVID-19 care units: a clinical trial. Holist Nurs Pract.

[25] Thimmapuram J (2021). Heartfulness meditation improves loneliness and sleep in physicians and advance practice providers during COVID-19 pandemic. Hosp Pract.

[26] Kisely S (2020). Occurrence, prevention, and management of the psychological effects of emerging virus outbreaks on healthcare workers: rapid review and meta-analysis. BMJ.

[27] Ghawadra SF (2019). Mindfulness-based stress reduction for psychological distress among nurses: a systematic review. J Clin Nurs.

[28] Sarazine J (2021). Mindfulness workshops effects on nurses’ burnout, stress, and mindfulness skills. Holist Nurs Pract.

[29] Muller AE (2020). The mental health impact of the covid-19 pandemic on healthcare workers, and interventions to help them: A rapid systematic review. Psychiatry Res.

[30] Murashiki D (2021). Which wellbeing resources are helpful in managing stress during Covid-19? Nursing. Times.

