# Supplementary material for: Psychosocial interventions for healthcare workers during the COVID-19 pandemic: rapid review and meta-analysis
Source: Wien Med Wochenschr. 2023 May 16;174(3-4):79–86. doi: 10.1007/s10354-023-01013-9 (PMC10185947; doi:10.1007/s10354-023-01013-9)
Supplement: Supplementary file 1 — Supplementary Information Tables 1 to 7 Table 1. Search terms used for literature search Table 2. Search strings for the databases (exemplary for systematic reviews) Table 3. Excluded systematic reviews Table 4. AMSTAR quality assessment of the included systematic reviews Table 5. JBI quality assessment of included primary studies Table 6. Characteristics of the included studies Table 7. Results of the included studies according to stress-related outcomes [file 10354_2023_1013_MOESM1_ESM.docx]

*Title:* Psychosocial interventions for healthcare workers during the COVID-19 pandemic: Rapid review and meta-analysis

**Supplementary Informations**

Table 1. Search terms used for literature search

Table 2. Search strings for the databases (exemplary for systematic reviews)

Table 3. Excluded systematic reviews

## Table 4: AMSTAR quality assessment of the included systematic reviews

Table 5: JBI quality assessment of included primary studies

Table 6: Characteristics of the included studies

Table 7: Results of the included studies according to stress-related outcomes

Supplementary Information A: search strategy

Table 1. Search terms used for literature search

| **Intervention** | **Outcome** | **Population** |
| --- | --- | --- |
| **Psychosocial interventions:** Psychosocial interventions, psychosocial measures, mental health measures, supervision, psychotherapy, cognitive- behavioural therapy, support, training, occupational therapy | **Outcome:** Stress, depression, anxiety disorder, sleep disorder, sleep disturbance, insomnia, burnout, distress, anxiety, psychological distress  **COVID-19:** Coronavirus, SARS-CoV-2, Corona, COVID-19, 2019-novel-corona*, 2019-new-corona, novel-corona, new-corona, 2019-CoV, 2019-nCoV, nCoV, coronavirus disease-2019, SARS2, SARS-2, 2019ncov, coronavirus-2 | **Healthcare workers:** nursing, nursing personnel, nursing staff, healthcare worker, health personnel, personnel, hospital, health provider*, medical worker*, medical personnel, medical professional*, nurses, nurses’ aides, HCW* |

##

Table 2. Search strings for the databases (exemplary for systematic reviews)

| **Database** | **Search string** |
| --- | --- |
| Cochrane Database of Systematic Reviews, Epistemonikos | #1 MeSH descriptor: [Social Support] explode all trees  #2 MeSH descriptor: [Mental Health Services] explode all trees  #3 MeSH descriptor: [Psychotherapy] explode all trees  #4 MeSH descriptor: [Occupational Therapy] explode all trees  #5 MeSH descriptor: [Psychiatric Rehabilitation] explode all trees  #6 ((Psychosocial OR psychological OR psycho*) AND (measure OR training OR intervention)) OR (mental health measure) OR supervision OR superv* OR psychotherapy OR (cognitive-behavioural therapy) OR (cognitive behavioural therapy) OR (psychological support) OR (psychosocial support) OR (occupational therapy)  #7 #1 OR #2 OR #3 OR #4 OR #5 OR #6  #8 MeSH descriptor: [COVID-19] explode all trees  #9 MeSH descriptor: [SARS-CoV-2] explode all trees  #10 Coronavirus OR SARS-CoV-2 OR Corona OR Covid-19 OR novel-corona OR new-corona OR nCoV OR SARS2 OR SARS-2 OR coronavirus-2 5746  #11 #8 OR #9 OR #10  #12 Nurs* OR nursing personnel OR nursing staff OR healthcare worker OR health personnel OR health provider OR medical worker OR medical personnel OR medical professional  #13 MeSH descriptor: [Health Personnel] this term only  #14 MeSH descriptor: [Nurse Practitioners] explode all trees  #15 #12 OR #13 OR #14  #16 Stress AND Depression AND (anxiety disorder) OR (sleep disorder) OR sleep disturbance OR insomnia OR burnout OR distress OR anxiety OR psychological AND distress  #17 MeSH descriptor: [Stress, Psychological] explode all trees  #18 MeSH descriptor: [Stress, Physiological] explode all trees  #19 MeSH descriptor: [Depression] explode all trees  #20 MeSH descriptor: [Depressive Disorder] explode all trees  #21 MeSH descriptor: [Anxiety] explode all trees  #22 MeSH descriptor: [Anxiety Disorders] explode all trees  #23 MeSH descriptor: [Sleep Wake Disorders] explode all trees  #24 MeSH descriptor: [Mental Fatigue] explode all trees  #25 #17 OR #18 OR #19 OR #20 OR #21 OR #22 OR #23 OR #24  #26 #7 AND #11 AND #15 AND #25 |
| PubMed | #14  Search: #12 AND #9 AND #6 AND #3 Filters: Clinical Trials  #13  Search: #12 AND #9 AND #6 AND #3 Filters: Systematic Review  #12  Search: #10 OR #11  #11  Search: health personnel[MeSH Terms]  #10  Search: (((((((((((((((((((((((((HCWs) OR (HCW)) OR (nurse)) OR (nurses)) OR (nurs*)) OR (medical professionals)) OR (medical professional)) OR (medical professional*)) OR (medical personnel*)) OR (medical personnel)) OR (medical workers)) OR (medical worker)) OR (medical worker*)) OR (health providers)) OR (health provider)) OR (health provider*)) OR (health personnel)) OR (health personnel*)) OR (healthcare worker*)) OR (healthcare workers)) OR (healthcare worker)) OR (nursing staff)) OR (nursing staff*)) OR (nursing personnel)) OR (nursing personnel*)) OR (nursing)  #9  Search: #7 OR #8  #8  Search: (((((((((((((((((((((((psychological distress*) OR (psychological distress)) OR (anxiety)) OR (anxieties)) OR (anxiet*)) OR (distress*)) OR (distress)) OR (burnout*)) OR (burnout)) OR (insomnia*)) OR (insomnia)) OR (sleep disturbances)) OR (sleep disturbance)) OR (sleep disturbance*)) OR (sleep disorders)) OR (sleep disorder)) OR (sleep disorder*)) OR (anxiety disorders)) OR (anxiety disorder)) OR (anxiety disorder*)) OR (depress*)) OR (depression)) OR (stress)) OR (stress*)  #7  Search: (((((((psychological stress[MeSH Terms]) OR (physiological stress[MeSH Terms])) OR (depression[MeSH Terms])) OR (depressive disorder[MeSH Terms])) OR (anxiety[MeSH Terms])) OR (anxiety disorders[MeSH Terms])) OR (sleep wake disorders[MeSH Terms])) OR (mental fatigue[MeSH Terms])  #6  Search: #4 OR #5  #5  Search: ((((((((((((((((((((((coronavirus-2) OR (2019ncov)) OR (SARS-2)) OR (SARS2)) OR (coronavirus disease-2019)) OR (nCoV)) OR (2019-nCoV)) OR (2019-CoV)) OR (new-corona*)) OR (new-corona)) OR (novel-corona*)) OR (novel-corona)) OR (2019-new-corona))) OR (2019-new-corona*)) OR (2019-novel-corona)) OR (2019-novel-corona*)) OR (covid-19)) OR (Corona)) OR (SARS CoV-2)) OR (coronavirus*)) OR (Coronaviruses)) OR (Coronavirus)  #4  Search: (COVID-19[MeSH Terms]) OR (SARS-CoV-2[MeSH Terms])  #3  Search: #1 OR #2  #2  Search: (((occupational therapy[MeSH Terms]) OR (psychotherapy[MeSH Terms])) OR (social support[MeSH Terms])) OR (mental health services[MeSH Terms])  #1  Search: ((((((((((((((((((((((((((occupational therapy) OR (occupational therapies)) OR (occupational therap*)) OR (training)) OR (trainings)) OR (training*)) OR (support)) OR (support*)) OR (cognitive-behavioural therapies)) OR (cognitive-behavioural therapy)) OR (cognitive-behavioural therap*)) OR (psychotherapies)) OR (psychotherapy)) OR (psychotherap*)) OR (supervision)) OR (supervisions)) OR (supervision*)) OR (mental health measure)) OR (mental health measures)) OR (mental health measure*)) OR (psychosocial measure))) OR (psychosocial measures)) OR (psychosocial measure*)) OR (psychosocial intervention)) OR (psychosocial interventions)) OR (psychosocial intervention*) |
| CINAHL | S49 (S3 AND S6 AND S25 AND S45) AND (systematic review or meta-analysis or literature review or review of literature) NOT TI(rct or randomized control trial or randomized controlled trial or controlled trial or cohort or case control)  S48 S3 AND S6 AND S25 AND S45  S47 S3 AND S6 AND S25 AND S45  S46 S3 AND S6 AND S25 AND S45  S45 S33 OR S44  S44 S34 OR S35 OR S36 OR S37 OR S38 OR S39 OR S40 OR S41 OR S42 OR S43  S43 "anxiety"  S42 "psychological distress"  S41 "distress"  S40 "burnout"  S39 "insomnia"  S38 "sleep disturbance"  S37 "sleep disorder"  S36 "anxiety disorder"  S35 "depression"  S34 "stress"  S33 S26 OR S27 OR S28 OR S29 OR S30 OR S31 OR S32  S32 MH "anxiety disorder*"  S31 MH "anxiety"  S30 MH "depression"  S29 MH "professional burnout"  S28 MH "sleep disorder*"  S27 MH "occupational psychology"  S26 MH "occupational health"  S25 S11 OR S24  S24 S12 OR S13 OR S14 OR S15 OR S16 OR S17 OR S18 OR S19 OR S20 OR S21 OR S22  S23 ("occupational therapy") AND (S12 OR S13 OR S14 OR S15 OR S16 OR S17 OR S18 OR S19 OR S20 OR S21 OR S22)  S22 "occupational therapy"  S21 "training"  S20 "support"  S19 "cognitive behavioural therapy"  S18 "cognitive behavioural therap*"  S17 "cognitive-behavioural therap*"  S16 "psychotherap*"  S15 "supervision"  S14 "mental health measures"  S13 "psychosocial measures"  S12 "psychosocial interventions"  S11 S7 OR S8 OR S9 OR S10  S10 MH "stress management"  S9 MH "psychosocial rehabilitation"  S8 MH "mental health services"  S7 MH psychotherapy  S6 S4 OR S5  S5 ((MH "Nurses") OR (MH "Health Personnel") OR (MH "Medical Staff") OR (MH "Medical Staff, Hospital") OR (MH "Nursing Staff, Hospital") )  S4 "Nurs*" OR "nursing personnel*" OR "nursing staff*" OR "healthcare worker*" OR "health personnel*" OR "health provider*" OR "medical worker*" OR "medical personnel*" OR "medical professional*" OR "health care professional*" OR "nurses*" OR "nurses aides*" OR "HCW*"  S3 S1 OR S2  S2 ((MH "COVID-19") OR (MH "SARS Virus") OR (MH "Severe Acute Respiratory Syndrome") OR (MH "COVID-19") OR (MH "SARS Virus") OR (MH "Severe Acute Respiratory Syndrome") )  S1 ("Coronavirus*" OR "SARS CoV-2*" OR "Corona*" OR "Covid-19" OR "2019-novel-corona*" OR "novel-corona*" OR "new-corona*" OR "2019-CoV*" OR "2019-nCoV OR nCoV*" OR "coronavirus disease*" OR "SARS2" OR "SARS-2" OR "2019ncov" OR "coronavirus-2*")) |

Table 3. Excluded systematic reviews

| **Reasons for exclusion** | | | | |
| --- | --- | --- | --- | --- |
|  | **Design not fitting** | **Intervention not fitting** | **Endpoint not fitting** | **Reasons for exclusion** |
| Cabarkapa et al. 2020 | **1** | 0 | 0 | Design not fitting |
| D'Ettore et al. 2021 | 0 | **1** | 0 | Intervention not fitting |
| Dincer & Inangilb 2021 | 0 | **1** | 0 | Intervention not fitting |
| Drissi et al. 2020 | **1** | 0 | 0 | Design not fitting |
| Gross et al. 2021 | 0 | **1** | 0 | Intervention not fitting |
| Labrague et al.2021 | 0 | 0 | **1** | Endpoint not fitting |
| **Total** | **2** | **3** | **1** |  |

## Table 4: AMSTAR quality assessment of the included systematic reviews

| **Author** | **Question and inclusion** | **Protocol** | **Study design** | **Comprehensive search** | **Study selection** | **Data extraction** | **Excluded studies: justification** | **Included studies: details** | **Risk of bias** | **Funding sources** | **Conflict of interest** | **Total** |
| --- | --- | --- | --- | --- | --- | --- | --- | --- | --- | --- | --- | --- |
| **Kisley et al. 2020** | Yes | No | No | Partial Yes | Yes | Yes | Partial Yes | Yes | Yes | No | Yes | **8** |
| **Muller et al. 2020** | Yes | Partial Yes | Yes | Yes | No | Yes | Partial Yes | Yes | Yes | No | Yes | **9** |
| **Pollock et al. 2020** | Yes | Yes | Yes | Yes | Yes | Yes | Yes | Yes | Yes | Yes | Yes | **11** |
| **Zace et al. 2020** | Yes | Yes | Yes | No | Yes | Yes | Partial Yes | Partial Yes | Yes | No | Yes | **9** |

**Table 5: JBI quality assessment of included primary studies**

|  |  | **Fiol DeRoque et al. (2021)** | **Nourian et al. (2021)** | | **Thimmapuram et al. (2021)** | |
| --- | --- | --- | --- | --- | --- | --- |
| **1.** | **Was true randomization used to assign participants to treatment groups?** | Yes | | Yes | | Yes |
| **2.** | **Was allocation to treatment groups concealed?** | Yes | | No | | No |
| **3.** | **Were treatment groups similar at the baseline?** | Yes | | Unclear | | Yes |
| **4.** | **Were participants blind to treatment assignment?** | Yes | | No | | No |
| **5.** | **Were those delivering treatment blind to treatment assignment?** | Unclear | | No | | No |
| **6.** | **Were outcome assessors blind to treatment assignment?** | Yes | | No | | No |
| **7.** | **Were treatment groups treated identically other than the intervention of interest?** | Yes | | No | | Yes |
| **8.** | **Was follow-up complete and, if not, were differences between groups in terms of their follow-up adequately described and analysed?** | Yes | | No | | No |
| **9.** | **Were participants analysed in the groups to which they were randomized?** | Yes | | No | | No |
| **10.** | **Were outcomes measured in the same way for treatment groups?** | Yes | | Yes | | Yes |
| **11.** | **Were outcomes measured reliably?** | Yes | | Yes | | Yes |
| **12.** | **Was an appropriate statistical analysis method used?** | Yes | | Yes | | Unclear |
| **13.** | **Was the trial design appropriate and were any deviations from the standard RCT design (individual randomization, parallel groups) accounted for in the conduct and analysis of the trial?** | Yes | | Yes | | Yes |
|  | **Total:** | **12/13** | | **5/13** | | **6/13** |

**Table 6: Characteristics of the included studies**

| Authors (Year) | Study design | Characteristics of the study | | | | | Quality of the study (tool and results) |
| --- | --- | --- | --- | --- | --- | --- | --- |
|  |  | Healthcare workers and population size | Type of setting | Intervention type | Intervention details | Outcome measures: instruments |  |
| Fiol-DeRoque et al. (2021) | RCT | Healthcare professionals *n* = 482 | Hospitals, home care centres, professional associations, scientific societies and trade unions | PsyCovidApp | Psychoeducational, self-guided app which focuses on increasing mindfulness in HCWs dealing with COVID-19, without the support of a therapist.  IG had access to the app for 14 days.  CG had access to the “Clinicovery” app, which provided info on the topic of mental health of HCWs. | - Depression, Anxiety, and Stress Scale (DASS-21) - Davidson Trauma Scale - Maslach Burnout Inventory (MBI-HSS) - Insomnia Severity Index (ISI) | JBI Checklist for RCTs: 12/13 |
| Nourian et al. (2021) | RCT | Nurses *n* = 41 | Hospital | Mindfulness-based stress reduction program (MBSR) | IG performed the MBSR program for 7 weeks. This included audio files for meditation, video instructions for yoga exercises, and audio and video files with professionals providing instruction on how to perform mindfulness exercises and texts on the nature of mindfulness.  CG were provided with music or training items with regard to caring for COVID-19 patients. | - Pittsburgh   Sleep Quality Index (PSQI) | JBI Checklist for RCTs: 5/13 |
| Thimmapuram et al. (2021) | RCT | Physicians and advanced practice providers  *n* = 155 | Hospital | Heart-based meditation | IG listened to an audio-guided, heart-based meditation for 12 minutes in the morning (to encourage relaxation and meditation) and in the evening (to improve the sense of self).  CG had no intervention and were told to continue their daily routines as normal. | - Pittsburgh Sleep Quality Index (PSQI) | JBI Checklist for RCTs: 6/13 |
| Zhou et al. (2020) | Before and after | Nurses *n* = 71 | Hospital (emergency isolation ward for COVID-19 patients) | Personalized emergency training and psychological  support with mindfulness-based stress reduction | Training was online and on-site. It focused on guidelines for the treatment and diagnosis of COVID-19 as well as hospital guidelines and info on the handling of PPE.  Nurses received online counselling, along with on-site psychological support and mindfulness decompression therapy. | - self-rating anxiety scale (SAS) - self-rating depression scale (SDS) | JBI – quasi experimental study: 7/7 |

Abbreviations: IG= intervention group; CG= control group, HCW= health care workers; DASS-21= Depression, Anxiety, Stress Scale; DTS= Davidson Trauma Scale; ISI=Insomnia Severity Index; MBI-HSS = Maslach Burnout Inventory-Human Services Survey; SAS= self-rating anxiety scale; SDS= self-rating depression scale; PSQI= Pittsburgh Sleep Quality Index, PPE= protective personal equipment, JBI= Joanna Briggs Institute

**Table 7: Results of the included studies according to stress-related outcomes**

| **Intervention (Authors)** | **Psychological Distress/PTSD** | **Anxiety** | **Burnout** | **Depression** | **Insomnia/Sleep** |
| --- | --- | --- | --- | --- | --- |
| PsyCovidApp (Fiol-DeRoque et al. 2021) | No significant decrease (DASS-21 stress subscale): *p* = 0.05  No significant decrease in posttraumatic stress (DTS): *p* = 0.47 | No significant decrease (DASS-21 anxiety subscale):  *p* = 0.17 | No significant improvement in emotional exhaustion (MBI-HSS subscale): *p* = 0.39  No significant improvement in professional accomplishment (MBI-HSS subscale): *p* = 0.12  No significant improvement in depersonalization (MBI-HSS subscale): *p* = 0.36 | No significant improvement (DASS-21 depression subscale): *p* = 0.47 | No significant decrease of Insomnia (ISI): *p* = 0.38 |
| Mindfulness-based stress reduction program (Nourian et al. 2021) |  |  |  |  | No significant improvement in overall sleep quality (PSQI): *p* = 0.105  Significant improvement in subjective sleep quality (PSQI subscale): *p* = 0.000  Significant improvement in sleep latency (PSQI subscale): *p* = 0.020  No significant improvement in habitual sleep efficiency (PSQI subscale): *p* = 0.148  No significant improvement in use of sleep medication (PSQI subscale): *p* = 0.118  No significant improvement in daytime drowsiness (PSQI subscale): *p* = 0.05 |
| Heart-based meditation (Thimapuram et al. 2021) |  |  |  |  | Significant improvement in Sleep Quality (PSQI) in intervention group post intervention: *p* = 0.001 |
| Personalized emergency training and psychological  support with mindfulness-based stress reduction (Zhou et al. 2020) |  | Significant reduction post-intervention (SAS): *p* = 0.019 |  | No significant reduction post-intervention (SDS): *p* = 0.306) |  |

Abbreviations: DASS-21= Depression, Anxiety, Stress Scale; DTS= Davidson Trauma Scale; ISI=Insomnia Severity Index; MBI-HSS= Maslach Burnout Inventory-Human Services Survey; SAS= self-rating anxiety scale; SDS= self-rating depression scale; PSQI= Pittsburgh Sleep Quality Index
